# Aplasie cutanée circonscrite du cuir chevelu chez un enfant rwandais: a propos d'une observation

**DOI:** 10.11604/pamj.2014.19.226.5096

**Published:** 2014-10-29

**Authors:** Olga Ntumba-Tshitenge, Célestin Kaputu-Kalala-Malu, Caritas Mukampunga, Kavulu Mayamba Mukendi

**Affiliations:** 1 Department of Pediatrics, Butare University Teaching Hospital, University of Rwanda, Rwanda; 2 Service of Child Neurology, Department of Neurology, Kinshasa School of Medicine, University of Kinshasa, Democratic Republic of Congo; 3 Centre Hospitalier Régional (CHR) Mons-Hainaut (MONS) et Cliniques Universitaires Saint Luc, Université Catholique de Louvain (UCL), Belgique, Département de Pédiatrie, Université de Lubumbashi (UNILU, RD Congo)

**Keywords:** Aplasie, cuir chevelu, malformation, Rwanda, Afrique, Aplasia, scalp, malformation, Rwanda, Africa

## Abstract

L'aplasie cutanée circonscrite du cuir chevelu ou aplasia cutis congenita, en nomenclature latine, est une malformation rare du cuir chevelu caractérisée par une absence de ce dernier. Elle peut intéresser l’épiderme ou toute la peau. La prise en charge d'une telle malformation est assez délicate. Elle est généralement chirurgicale et le pronostic dépend en grande partie de malformations vasculaires associées. Le risque infectieux et/ou hémorragique est élevé. Cette malformation peut être isolée, ne se limitant qu’à la peau du cuir chevelu; tout comme elle peut faire partie d'un syndrome polymalformatif associant l'atteinte d'autres tissus ou organes tel qu'on peut l'observer dans le syndrome d'Adams-Oliver. Nous rapportons le cas d'un nouveau-né rwandais chez qui on a constaté, à la naissance, une absence du cuir chevelu, parcellaire, intéressant une grande partie du vertex fronto-pariétal et la région pariétale gauche. Une IRM avec séquences angiograpiques réalisée plusieurs jours plus tard, montre une agénésie de la partie frontale du sinus longitudinal supérieur. A notre connaissance, aucune observation faisant état de cette malformation n'a jamais été publiée en Afrique Centrale.

## Introduction

Les malformations de l'ectoderme embryonnaire intéressent les structures dérivant de ce feuillet. Elles constituent un groupe hétérogène d'environ 200 maladies héréditaires décrites sous l'appellation de dysplasies ectodermiques dont les premiers cas ont été décrits par Thurmann en 1848 [1]. L'aplasie du cuir chevelu est presque toujours associée à ces différents syndromes polymalformatifs, entre autres le syndrome d'Adams-Oliver où l'aplasia cutis congenita du cuir chevelu est associée aux malformations des extrémités des membres et à un trouble de croissance, les aberrations chromosomiques, le syndrome de Bart et le syndrome de Johanson-Blizzard [2, 3]. Les formes dans lesquelles l'aplasie cutanée du cuir chevelu se présente de manière tout à fait isolée ont été décrites par certains auteurs [4]. Le diagnostic clinique est généralement évoqué à la naissance lorsque le pédiatre constate une absence partielle du cuir chevelu, pouvant également intéresser les os du crâne sous-jacent, la dure mère ou l'encéphale sous la forme d'une encéphalocèle [5]. Le traitement peut être conservateur et ceci concerne les cas d'aplasie simple du cuir chevelu, soit chirurgical dans le cas de malformations associées, osseuse, dure-mérienne ou encéphalique [6]. On rencontre parfois une malformation du sinus longitudinal supérieur, partielle ou complète, pouvant donner lieu à une circulation veineuse collatérale se présentant sous la forme de veines turgescentes et tortueuses au niveau du cuir chevelu ou même d'une hémorragie du sinus longitudinal supérieur rendant la prise en charge délicate [7]. Nous rapportons le cas d'un enfant rwandais chez qui on a constaté une aplasie circonscrite du cuir chevelu intéressant en grande partie le cuir chevelu du vertex fronto-pariétal et, en moindre mesure, celui de la région pariétale gauche. Nous avons également remarqué des veines turgescentes et tortueuses du cuir chevelu et l'angio-IRM, réalisée quelques jours plus tard, a mis en évidence une aplasie de la partie frontale du sinus longitudinal supérieur rarement décrites dans cette malformation. Les os du crâne sont intacts.

## Patient et observation

Il s'agit d'un nouveau-né du sexe féminin, née d'une mère de 30 ans sans problèmes. Elle est troisième d'une fratrie de 3 enfants. Le premier accouchement a été eutocique; le deuxième accouchement a eu lieu par césarienne pour dystocie dynamique et le troisième accouchement (la patiente) a eu lieu par césarienne itérative et élective, le 19/04/2014 à 39 SA. Le poids a été de 2500 g, la taille a été de 47 cm (P25-50) et le périmètre crânien (PC) a été mesuré à 35 cm (P50). L'APGAR a été de 9, 10, 10 et la température rectale à 36,6°, le pouls à 163/min, la SaO2 à 98% à l'air libre. La maman a bénéficié de 5 visites prénatales dont trois avec échographies. Elle n'a reçu aucun traitement médicamenteux pendant la grossesse dont le déroulement a été sans histoire. Sa sérologie VIH a été négative et elle est du groupe sanguin O (ABO).

**Figure 1:**
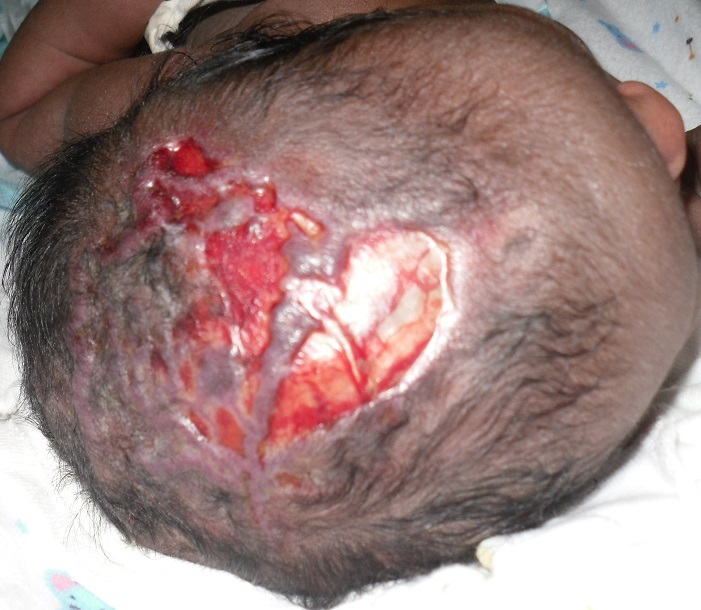
Absence du cuir chevelu dans les régions du vertex fronto-pariétal et médio-pariétale gauche au 1er jour de vie

**Figure 2:**
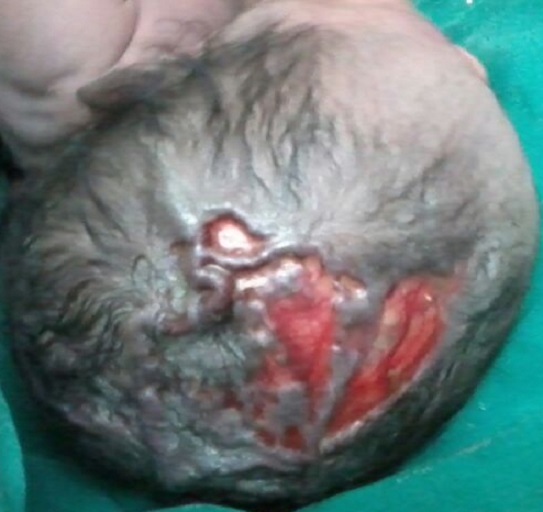
Des veines tortueuses et turgescentes traversant les zones malformées

L'enfant est envoyée dans le service de néonatologie pour prise en charge d'une plaie non traumatique du cuir chevelu. A son arrivée, l'examen général est bon (apparence, croissance et coloration normales; pas d'ictère). Le cri et l'auscultation cardiaque sont normaux et il n'y a pas de cyanose. Sur le plan de la circulation sanguine, le pouls est bien frappé, la peau est chaude. les bruits cardiaques, ajouter un s à bruit et au deuxième mot bruit qui suit. Les mouvements sont symétriques, le tonus est normal. Les réflexes archaïques sont normaux. L'ombilic, l'anus et la bouche sont normaux. Il n'a pas de malformation de la colonne vertébrales et pas de dysmorphie crânio-faciale. Par contre, on remarque une absence du cuir chevelu dans les régions du vertex fronto-pariétale et médio-pariétale gauche (Figure 1). On distingue nettement des veines turgescentes et tortueuses qui traversent les régions décrites ci-dessus et certaines autres la circonscrivent (Figure 2). A travers la grande fontanelle, on aperçoit, par transparence, la substance cérébrale.

**Figure 3:**
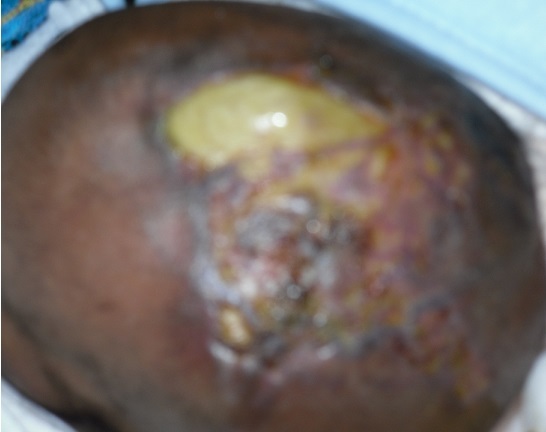
Zone de kératinisation de la région malformée commençant en vertex frontal, constatée au 10è jour de vie

**Figure 4:**
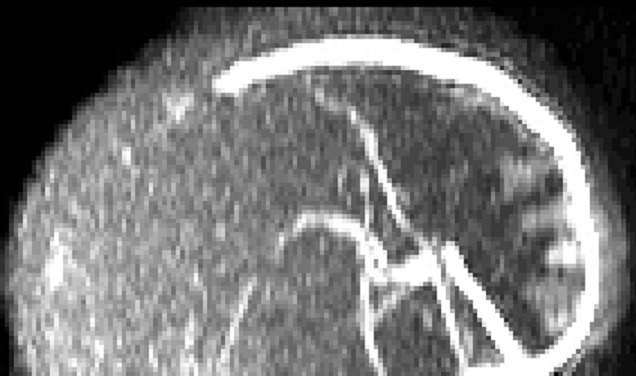
Image IRM réalisée au 15è jour de vie. Elle montre, en séquence agiographique, une agénésie de la partie frontale du sinus longitudinal supérieur

Quelques examens paracliniques ont été réalisés à l'admission, entre autres, l’échographie transfontanellaire qui était normale, une échographie abdominale, à la recherche de malformations associées, qui était également normale. Les analyses hématologiques sont normales; la glycémie et la créatinine sont normales. Les soins locaux ont été appliqués par pansement à l'acide fusidique. Par crainte d'une éventuelle surinfection, une couverture antibiotique a été appliquée par une association gentamicine et cloxacilline par voie veineuse. Au J 10, nous remarquons un début de kératinisation (Figure 3) et au J 15, une IRM de l'encéphale avec séquences angiographiques est réalisée et elle montre un cerveau de structure normale et une aplasie du sinus longitudinal supérieur dans son territoire frontal (Figure 4).

## Discussion

La malformation du cuir chevelu constatée chez cette fille pose un problème de prise en charge dans nos milieux compte tenu de l'infrastructure de la plupart des hôpitaux en Afrique Centrale. L'association à une aplasie partielle du sinus longitudinale supérieure avec circulation veineuse collatérale, comme nous l'avons constaté chez notre patiente, augmente les risques d'une élévation de la tension intracrânienne suite à l’élévation de la pression veineuse [8]. Elle augmente également les risques d'hémorragie par rupture des veines collatérales du cuir chevelu et/ou une hémorragie à départ du sinus longitudinal supérieur [7]. La recherche étiologique de ces malformations dépasse les moyens diagnostiques qu'aucun pays d'Afrique Central ne possède. Aucun antécédent familial de malformation n'a été signalé concernant notre patiente. Certaines étiologies sont évoquées dans la littérature entre autres les causes génétiques, les tératogènes, les infarctus placentaires, les infections fœtales, les dysplasies etc... [9]. Le diagnostic clinique est aisé, à la suite d'une constatation de perte de substance au niveau du cuir chevelu à la naissance ou plus tard, à la constatation d'une cicatrice (kératinisation) d'une partie plus ou moins étendue du cuir chevelu avec perte des cheveux.

## Conclusion

L'aplasie cutanée circonscrite du cuir chevelu est une malformation rare et de diagnostic clinique facile, mais de prise en charge difficile et surtout s'il s'y associe des malformations complexes comme décrites ci-dessus. Ce problème semble crucial dans les pays d'Afrique Subsaharienne. Notre observation montre à suffisance que de telles malformations existent en Afrique et qu'elles sont très probablement mal reconnues. L'examen clinique minutieux d'un nouveau-né et la connaissance parfaite des syndromes malformatifs peut faciliter leur diagnostic soit à la naissance soit un peu plus tard dans la vie de l'enfant porteur d'une malformation.
